# Highlights of Thirty-Year Experience of CO_2_ Laser Use at the Florence (Italy) Department of Dermatology

**DOI:** 10.1100/2012/546528

**Published:** 2012-04-19

**Authors:** Piero Campolmi, Paolo Bonan, Giovanni Cannarozzo, Andrea Bassi, Nicola Bruscino, Meena Arunachalam, Michela Troiano, Torello Lotti, Silvia Moretti

**Affiliations:** Section of Clinical, Preventive and Oncologic Dermatology, Department of Critical Care Medicine and Surgery, University of Florence, Villa S.Chiara, Piazza Indipendenza 11, 50129 Florence, Italy

## Abstract

The CO_2_ laser has been used extensively in dermatological surgery over the past 30 years and is now recognised as the gold standard for soft tissue vaporization. Considering that the continuous wave CO_2_ laser delivery system and the newer “superpulsed” and scanned CO_2_ systems have progressively changed our practice and patient satisfaction, a long range documentation can be useful. Our experience has demonstrated that the use of CO_2_ laser involves a reduced healing time, an infrequent need for anaesthesia, reduced thermal damage, less bleeding, less inflammation, the possibility of intra-operative histologic and/or cytologic examination, and easy access to anatomically difficult areas. Immediate side effects have been pain, erythema, edema, typically see with older methods, using higher power. The percentage of after-treatment keloids and hypertrophic scars observed was very low (~1%) especially upon the usage of lower parameters. The recurrence of viral lesions (condylomas and warts) have been not more frequent than those due to other techniques. Tumor recurrence is minor compared with radiotherapy or surgery. This method is a valid alternative to surgery and/or diathermocoagulation for microsurgery of soft tissues. Our results are at times not consistent with those published in the literature, stressing the concept that multicentric studies that harmonization methodology and the patient selection are vital.

## 1. Introduction

Laser surgery has become a powerful and indispensable instrument in dermatology. The first CO_2_ laser systems, which used continuous wave delivery systems, were efficient at ablating and also cutting the tissues. The high incidence of possible scars, however, limited its usage in the vaporization of thin, superficial layers. The efficacy was guaranteed, but the uninterrupted energy transfer often elicited a thermal damage extending in the tissue up to a depth of approximately 0.5–1 mm, carbonizations, frequent pigmentary modifications, and a slower cicatrization process. Technological development has yielded high-energy, “superpulsed,” and scanned CO_2_ systems which are now able to emit shorter pulses with high peaks of power. This in turn allows laser surgeons to ablate epidermal and dermal tissue with minimal risk of scarring, results in a precise and adequate vaporization of superficial skin layers, and limits thermal damage to surrounding tissues [1, 2]. 

Initial modifications in the continuous wave laser system has involved the electronic shuttering of the continuous beam of light to produce 0.1 to 1 second “pulse” duration at a consistent power. After these initial changes, “superpulsed” systems capable of attaining peak powers 2 to 10 times higher and pulse durations 10 to 100 times shorter than conventional models were developed [3]. 

 The carbon dioxide (CO_2_) laser has been used extensively in dermatological surgery departments and is currently recognized as the gold standard for vaporization of the soft tissues [4, 5]. In the following paper, we report our experience in comparison with data already published in this field [6–45].

## 2. A 30-Year Survey of CO_2_ Laser Surgery

**Figure 1:**
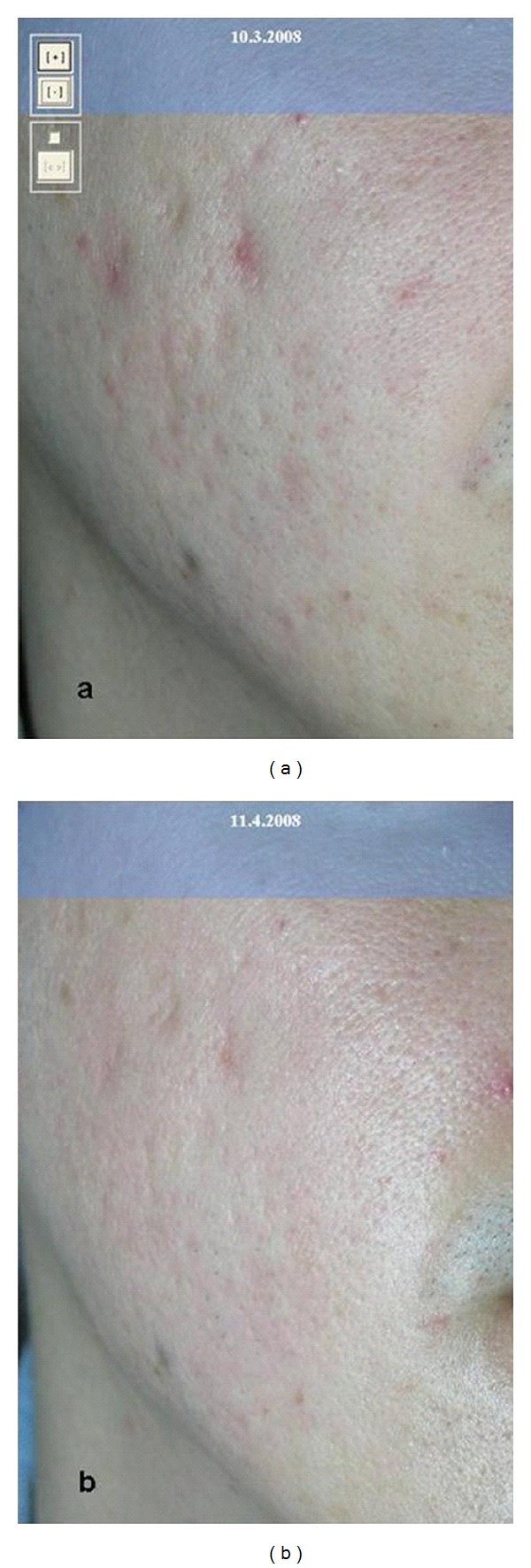
Acne scars at first (a) and after (b) only two treatments with fractional microablative CO_2_ laser.

**Figure 2:**
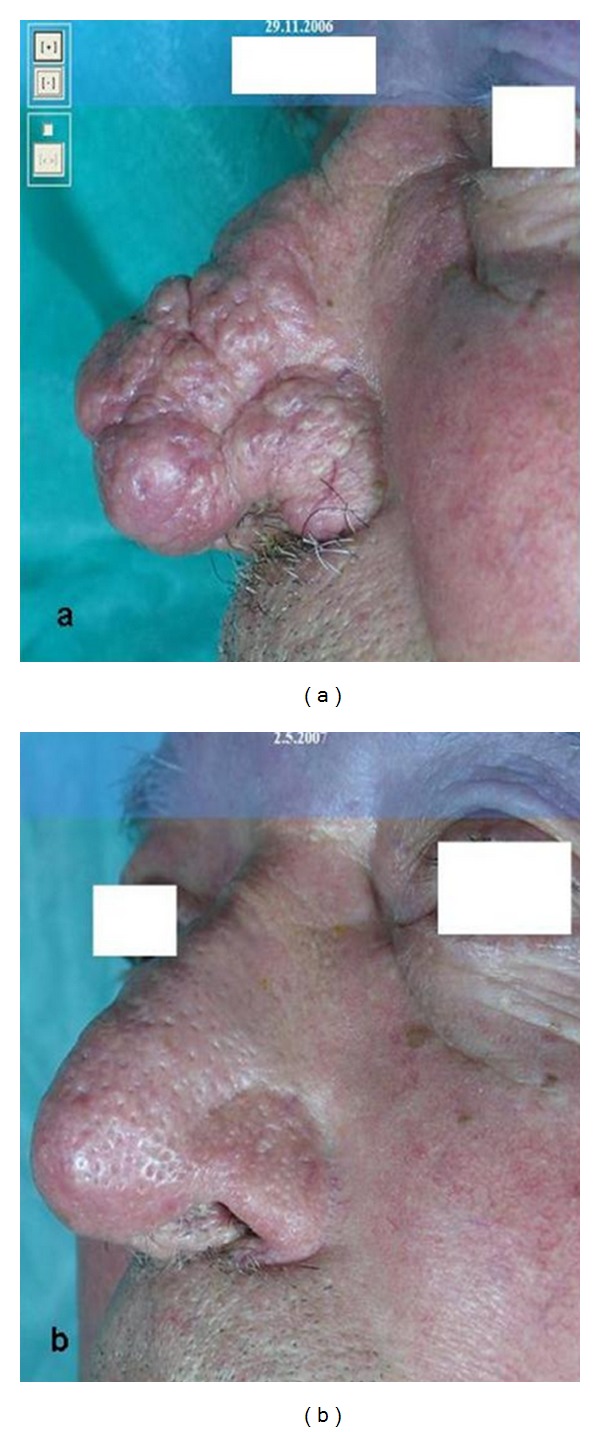
Rhinophyma at first (a) and after (b) five treatments with CO_2_ laser.

**Figure 3:**
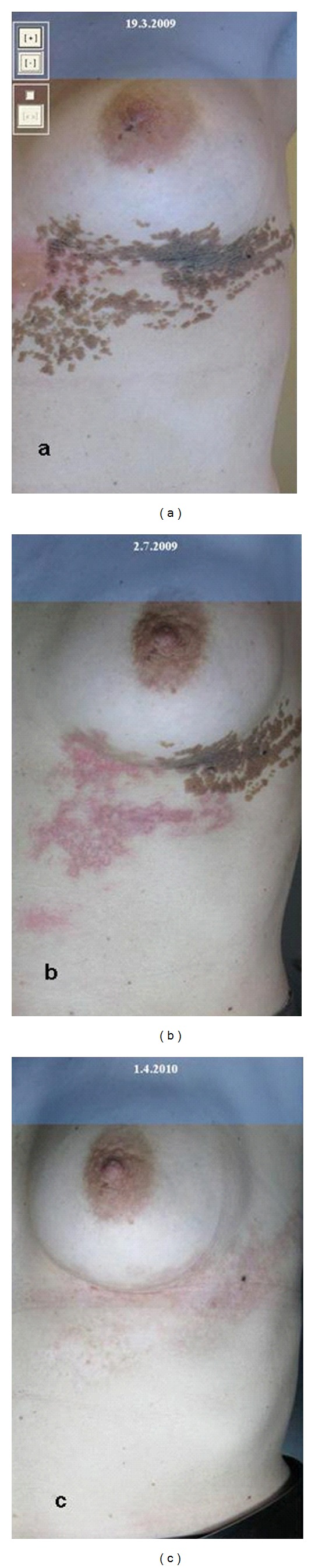
N. E. V. I. L. at first (a) and after three (b) and six (c) treatments with CO_2_ laser.

**Figure 4:**
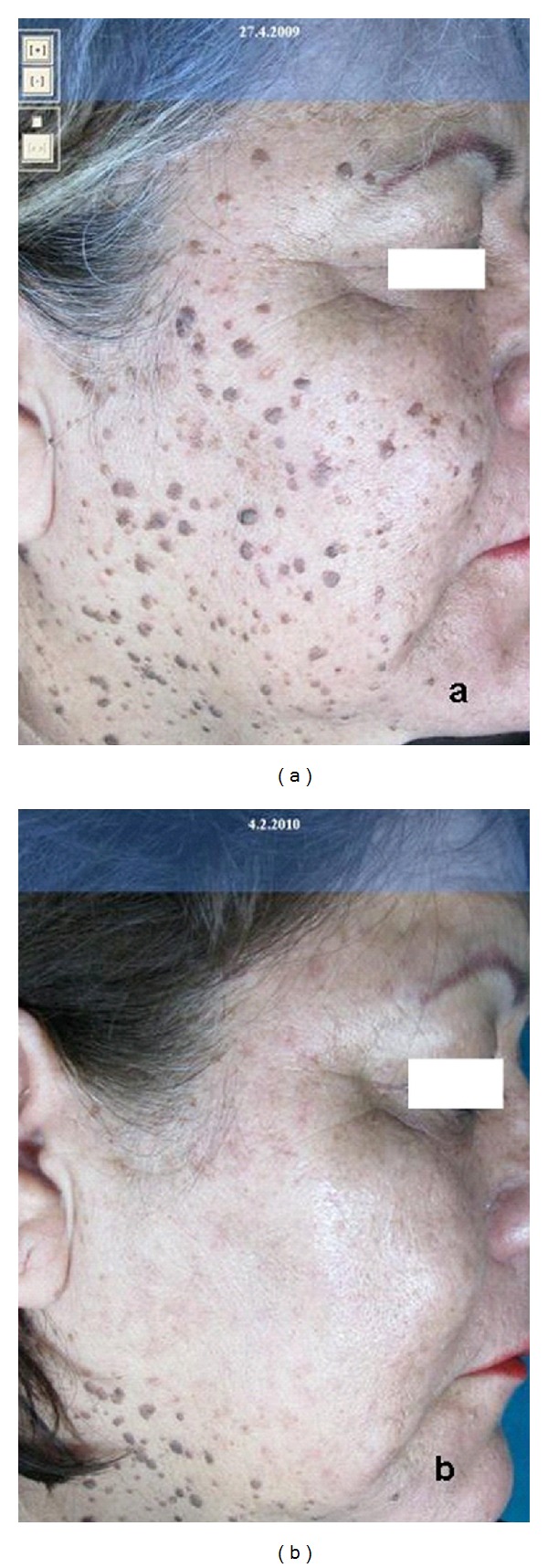
Dermatosis papulosa nigra at first (a) and after one (b) treatment with CO_2_ laser.

**Figure 5:**
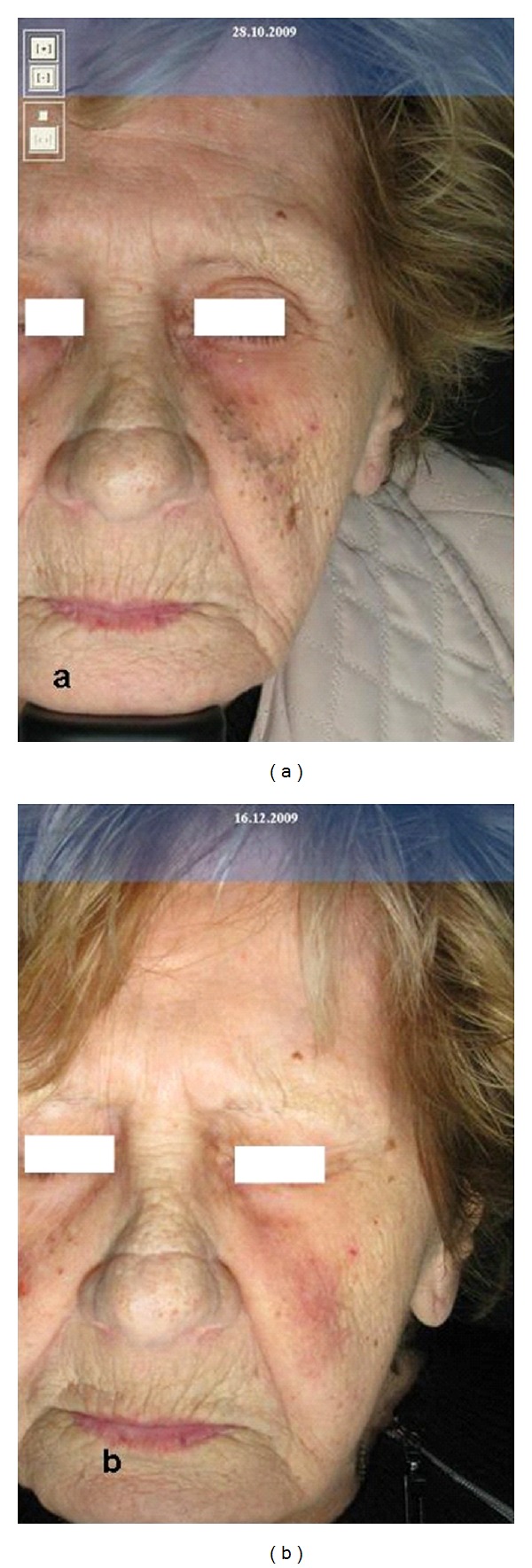
Favre-Racouchot's disease at first (a) and after (b) one treatment with CO_2_ laser.

**Figure 6:**
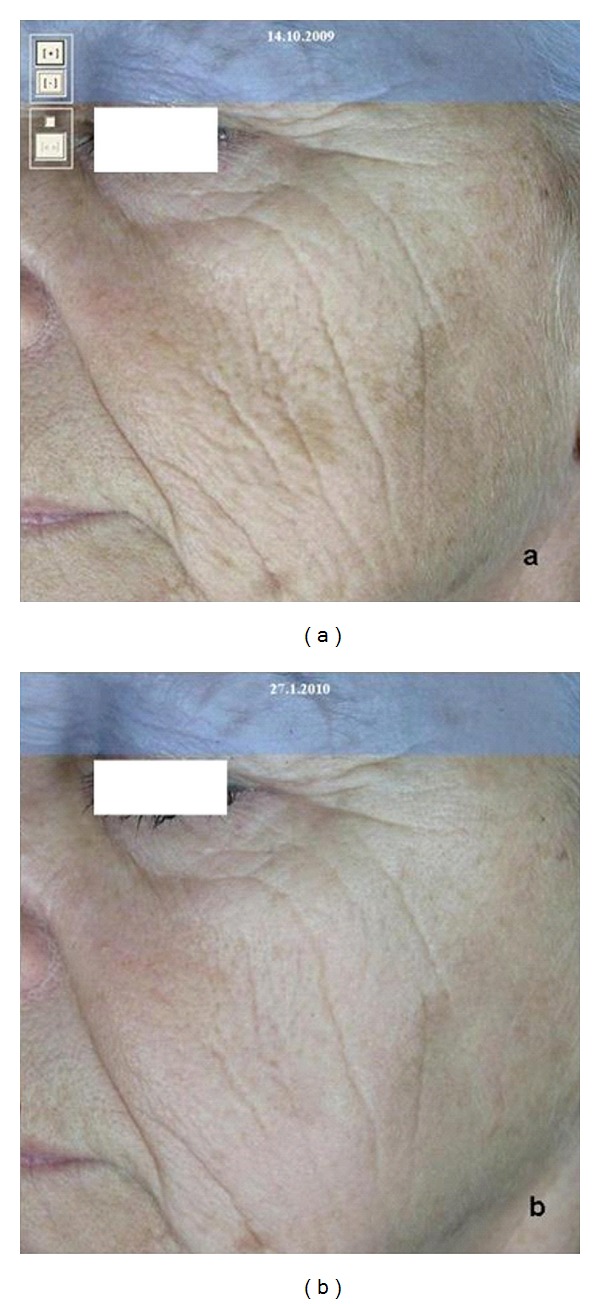
Facial wrinkles at first (a) and after (b) three treatments with fractional microablative CO_2_ laser.

**Figure 7:**
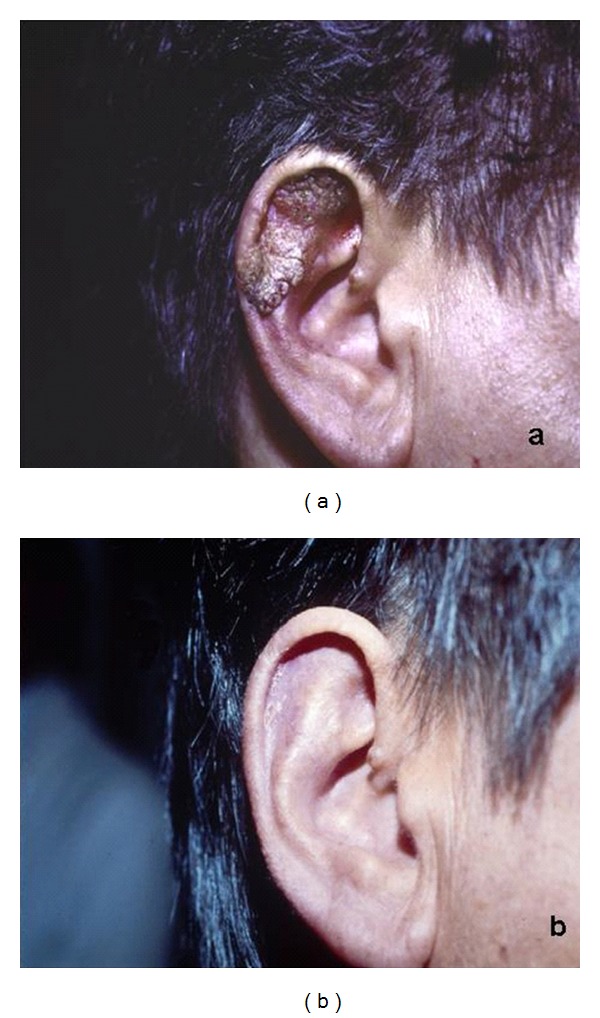
Vulgar warts at first (a) and after one (b) treatment with CO_2_ laser.

**Figure 8:**
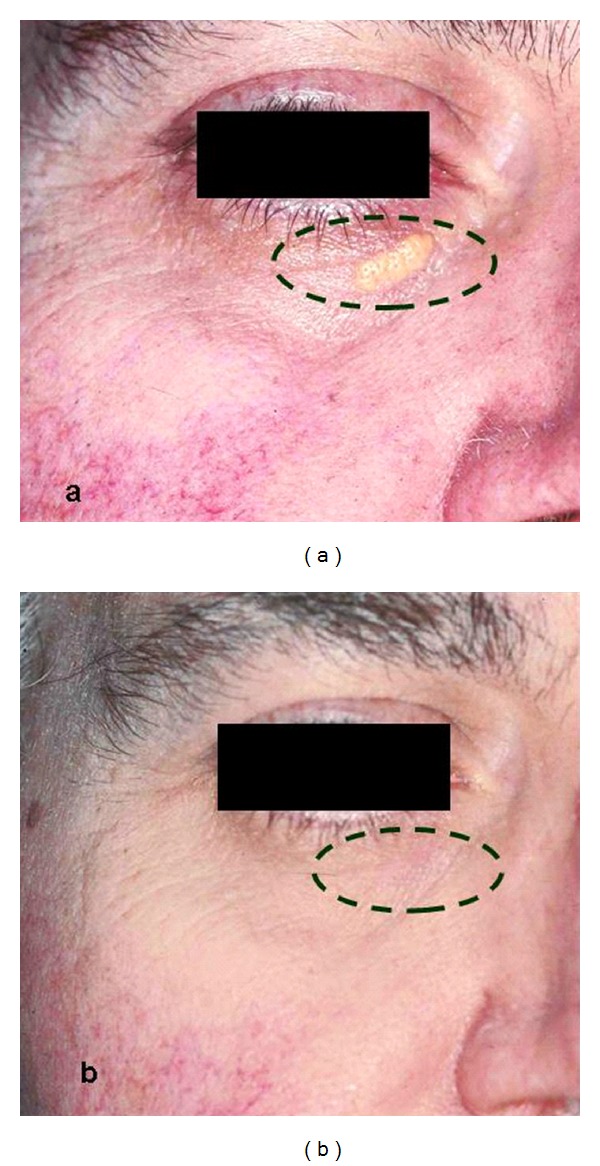
Xanthelasma at first (a) and after (b) one treatment with CO_2_ laser.

**Figure 9:**
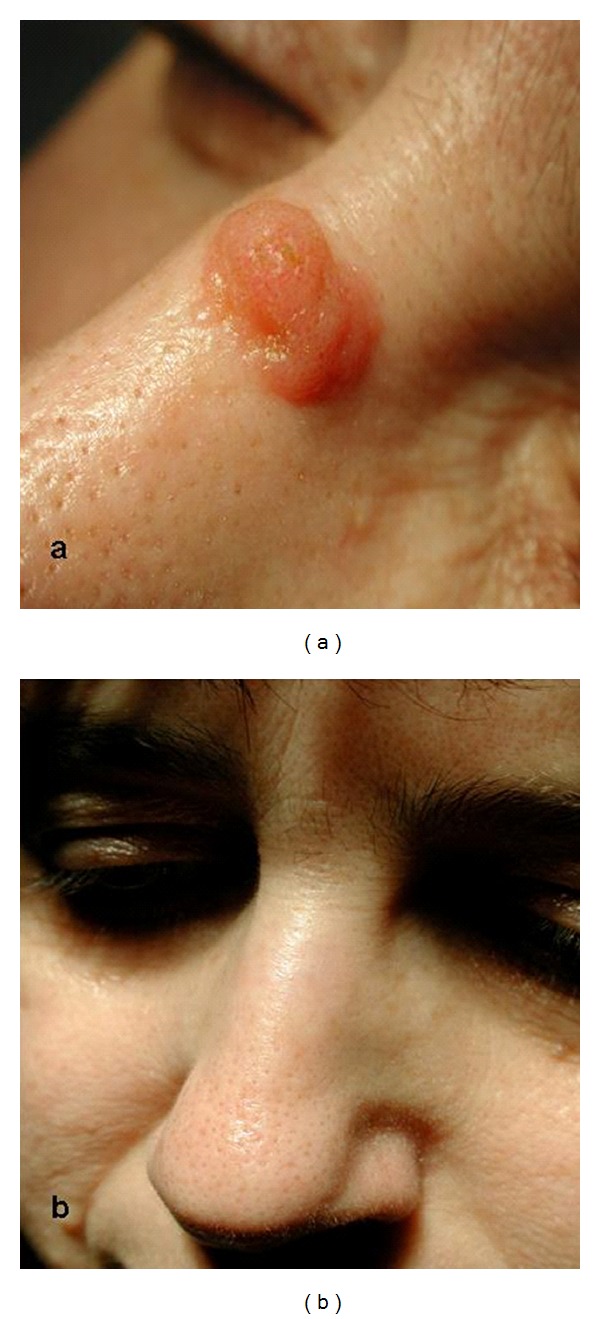
Inverted follicular keratosis at first (a) and after (b) one treatment with CO_2_ laser.

Given a thirty-year experience in the field of CO_2_ laser, we have surveyed a wide range of patients having various pathologies. In our clinic, 43,000 patients were treated, 18,000 males and 25,000 females. The age range was 20–90 years old, and approximately 300 patients were younger than 20. Common skin disorders such as molluscum contagiosum, palmar-plantar warts, and pyogenic granulomas were seen in our clinic (Figures 1, 2, 3, 4, 5, 6, 7, 8, and 9).

CO_2_ SmartXide laser (DEKA-M.E.L.A., Calenzano, Italy) is typically performed with a superpulse mode rather than a continuous one (except in cases of plantar warts, certain types of rhynophymas, and giant condylomas): this ensures that all treated zones are always “levelled” to the desired thickness. We usually operate with a power range of 0.5–1 W and a frequency of 10 Hz but sometimes increase the frequency to 20 Hz and use up to 2 W power. The actual values depend on the type of lesion; for example, with plantar warts we usually use a 10–15 W power in continuous mode and with *rhinophyma *we use a 50–100 HZ frequency with a 6–10 W power (Table 1).

Typically, we use slightly defocalized laser beams; however, for heating (i.e., seborrheic keratoses) or coagulation purposes, defocalized treatment is normally used.

An important advantage in the use of this type of laser is the reduced need for anaesthesia. In our clinical practice, we typically do not use anaesthesia with a power lower than 1-2 W and a frequency of 10–20 Hz. These parameters however depend upon the site, type, and level of lesions. We limit the use of anaesthesia since the procedure itself is less painful for the patient and also because local anaesthesia could cause edema and hinder the “visual feedback processing” during treatment. When treating more difficult and sensitive body areas, such as the eyes, periocular, oral, and anal areas, genital mucosa, and fingers, local anaesthesia is utilized. In such select cases, we sometimes suggest the application of a eutectic mixture of lidocaine 2.5% and prilocaine 2.5% in an oil-in-water emulsion, especially for small and superficial lesions. This is applied under occlusion two hours before treatment (thirty minutes for mucosal lesions) and removed just before the procedure. After vaporization, the carbonized residue is removed with gauze soaked in physiologic solution. In this way, “we can see immediately what we get.”

After treatment, we usually suggest the application of topical antibiotics on the lesion covered by a traditional patch, when bald areas, the face, or other photoexposed areas are involved. We use a hydrocolloid transparent dressing that is permeable to water vapour but impermeable to exudate and microorganisms since it has a high absorbency and can manage the wound exudate and skin secretions. The transparent dressing facilitates inspection of the wound without removing it; the healing process can therefore be monitored closely. The patient can remove the transparent dressing every day and wash the wound with a cleansing foam and then reapply the dressing, for one to two weeks, without crust formation, and protect the wound with sunscreens. The redness disappears within few weeks.

A major criticism regarding laser treatment for common skin neoplasms (e.g., in the treatment of basal cell carcinoma (BCC)), lies in the fact that this technique would not allow the operator to perform an intraoperative histopathologic/cytologic examination, thus limiting the certainty of its complete removal. In our practice, however, we have acquired extensive experience in treating BCCs with CO_2_ laser associated with intraoperative cytologic/histopathologic examination obtained by scraping the lesion prior to vaporising with laser through successive layers of the skin. The aforementioned technique is possible due to the use of low parameters, so as not to create thermal tissue damage. A similar procedure could be of interest for other skin diseases, such as actinic keratosis, squamous cell carcinoma (selective cases), actinic cheilitis, Bowen's disease, and leukoplakia, where intraoperative cytologic examination could be beneficial, as proposed by our laser team [46]. 

Another important novelty in the removal of wrinkles and damage caused by skin photoaging or other skin imperfections, such as acne scars, is the use of a modified device and associated software with a novel handpiece: the “fractional microablative resurfacing with CO_2_ laser” [47–58]. The introduction of scanner systems demonstrates a substantial progress in the field of skin resurfacing respect to CO_2_ laser demonstrated by free hand movement on the handpiece. Nevertheless the purpose of the traditional scanner with CO_2_ sources in resurfacing was connected with a higher occurrence of side effects due to intense heating and high thermal damage such as pain, long-lasting erythema, and pigmentary disorders [59]. With the advent of newer special scanning systems, the fractional CO_2_ laser emission takes place by means of dots (DOT), generating microareas of ablative and thermal damage (MTZ = microthermal zones) alternated with healthy tissue. In the treated microareas, a controlled heat release produces immediate tissue shrinkage and stimulates neocollagenogenesis. The areas of healthy tissue between the treated areas ensure rapid tissue repair and a drastic reduction in recovery time and posttreatment erythema [60–62]. This exclusive emission system allows us to control tissue damage while simultaneously maximizing efficacy. Thus, the fractional laser can be considered an effective alternative to topical treatments such as (Tretinoin, *α*-hydroxyacids), chemical peelings, botulinum toxin, and filler injections [63–66].

The settings usually implemented include a power of 10–30 W, a DOT spacing of 500–1000 *μ*m, stack 1–3, and dwell time of 500–2000 *μ*s. We usually prefer to work in a range of 10–20 W because it allows us to reach a lower intensity and duration of side effects. Anaesthetics are not needed in the procedure; rather external cryogen is utilized.

We must also consider that laser peels can activate a herpes virus infection and possibly other dormant pathogens. In most cases, a patient is given an oral antiviral drug or sometimes antibiotics in the case of bacterial infection before and after the laser surgery procedure. There is always a risk of postinflammatory pigmentary changes following any type of inflammatory process in the skin. Fractional laser treatments are not an exception—the risk is most common in patients with a history of postinflammatory hyperpigmentation (PIH) or melasma. PIH is more common in patients of darker skin types (IV–VI). A precautionary pre-post treatment with a lightening cream for a few weeks followed by a strict sun-protection regimen is advisable for all these individuals.

A new parameter is the stack setting. This function allows the physician to set the number of pulses to deliver on the same DOT of the tissue, before moving to the next DOT according to the scanning modality selected. For example, when choosing stack 3, the system automatically triples the number of pulses on the same DOT; thus the thermal and ablative effect on the tissue during a single passage are tripled, without a considerable change in the time required. This stack function multiplies the effect on the skin and is a much better approach than repeating a laser scan on the tissue three times (three “passages,” pushing three times the pedal of the laser device).

While using the latter traditional method, it is possible that the tissue can move during the session. In the next passage, the laser may not uniformly place itself above all the DOTs. The stack function, however, has a further advantage given by the quickness of the laser scanning [67–70]. In this manner, the ablation depth and stimulation of the tissues are precisely controlled, adapting the treatment to the area in question according to the specific skin condition and the patient's needs. This function allows deeper penetration since the laser treatment can be repeated with the same parameters over the dots in the same scanner passage, making possible a wide range of effects and further increasing its flexibility [70, 71]. 

## 3. Side Effects Experienced in Our Laser Practice

Laser treatment comes with the possibility of side effects. In our experience, immediate side effects consist of pain, erythema, and edema, which are found mainly with old methods, using higher powers, which result in long recovery times of weeks or even months. With the new “superpulsed” and scanned CO2 systems, which are associated with the use of lower parameters, we have been able to limit edema and erythema possibly within hours or days after treatment. In some cases, episodes of hypochromia and hyperchromia have taken place, which occur most often when treatments are carried out during the summer rather than winter. However, these have become transient with the use of newer instruments.

The percentage of after-treatment keloids and hypertrophic scars has been very low (~1%) above all when using lower parameters. The recurrence of viral lesions (condylomas and warts) have not been more frequent than those due to other techniques, and recurrence of the tumors has been minor compared with radiotherapy or surgery. Three patients developed retracting scars of the inner part of the upper eyelid after the treatment of xanthelasmas, while alopecic patches have developed in other five patients after the treatment of scalp lesions. We have seen ten cases of hypochromia and atrophy when CO_2_ laser was also used in the treatment of vascular lesions, such as venous gaps of the lips.

In regards to the use of fractional CO_2_ laser, a more prolonged erythema has resulted in dyschromia in our patients. Herpes virus reactivation or other infections did occur, even if our patients were treated with systemic antiviral or antibiotics before and after the treatment.

However, in addition to a correct setting of parameters by the dermatologist, equally important is the diligent medication after surgery that we recommend to our patients in order to avoid the occurrence of side effects that result in a more difficult and prolonged patient management.

## 4. Discussion

The basis of laser application is the conversion of laser energy into heat [72, 73]. The interaction between the electromagnetic radiation emitted by a laser source and biological tissues is governed by physical processes, which regulate the exchange of energy between the wave and the substrate, and by the biological response of the targeted tissue. Depending on the temperature reached in a specific area, the thermal energy produced is capable of coagulating, vaporizing, or ablating. With a sufficiently high fluence (above the minimum ablation threshold), the heat is mainly used for ablating or vaporising the targeted tissue, before beginning to spread more slowly towards the surrounding areas.

Thermal relaxation time (TRT) refers to the time it takes for the irradiated “target” tissue to lose 50% of the incident heat, without heat conduction to the surrounding tissues. The correct use of the surgical lasers takes into consideration that less thermal damage is obtained with higher power and laser pulses with shorter amplitudes than the TRT of the selected target. This is the theory behind selective photothermolysis, namely, selective tissue heating by preferential light absorption and heat production in the targeted tissue with appropriate wavelengths and pulse durations. Theoretical arguments suggest that using these principles combined with the proper pulse parameters would confine thermal damage and result in decreased scarring rates, improved clinical responses, and faster healing times [73–77].

 The CO_2_ laser works at a wavelength of 10600 nm, in the far-infrared spectrum where chromophore absorption of the intracellular and extracellular water molecules is prevalent.

**Figure 10:**
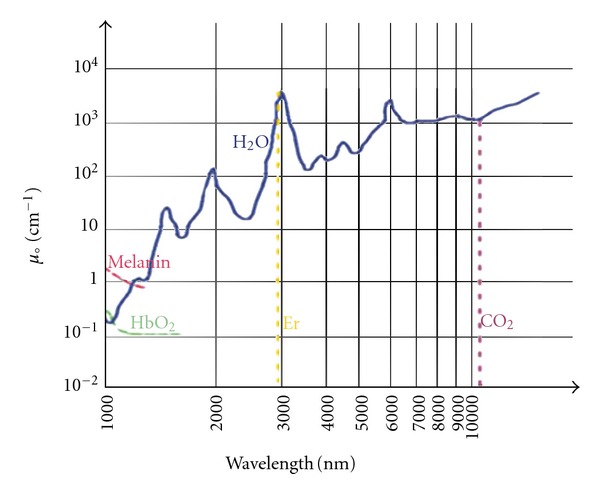
Water absorption coefficient in the infrared band.

 This high water absorption is an important factor that explains the reduced penetration depth of CO_2_ lasers, such as the Er: YAG laser whose penetration is much less than CO_2_ laser [78–82] (Figure 10). Water is the principal component of the skin (approximately 77% of its volume) and consequently plays an essential role in the laser-tissue interaction, above all in dermatology. CO_2 _ laser is a type of “no-contact surgery” belonging to the group of lasers in the category “WYSIWYG” (what you see is what you get—the operator can directly estimate the level reached during treatment with a “step-by-step” procedure). In fact, these laser systems enable precise, efficacious, and targeted thermal action on the lesions treated while at the same time protecting the adjacent areas thus guaranteeing optimal re-epithelialization. This makes it suitable for surgical procedures since the limited inflammatory response is conducive to better healing. The extreme precision of the application means that the epidermis alone can be vaporised, or the thermal effect can be extended even deeper into the papillary or reticular derma. Thanks to “colour indicators,” a step-by-step visual assessment of the level reached and an accurate calculation of the clinical “end-point” phase are possible. Clinical and histologic studies have demonstrated the effects of thermal damage in relation to the skin layer reached and the level of energy delivered. When vaporization is confined to the epidermis, an opalescent aspect is obtained, with the formation of microbubbles accompanied by a typical “crackling sound.” After removing the carbon residues with a physiologic solution, the papillary derma will appear and emerges as a flat, smooth, pink surface. Further vaporization reveals a hardened yellowish tissue similar to “chamois skin,” typical of the superficial derma. The next step, involving vaporisation of the reticular derma, reveals large collagen fibre fascias that in macroscopic terms look like “waterlogged cotton threads [2, 3, 73, 74].”

 The CO_2_ laser produces the most dramatic improvements in the clinical and histologic appearance of photodamaged or scarred skin. Epidermal ablation occurs after 1 pass of the CO_2_ laser at standard treatment parameters (vaporizing tissue to a depth of 20–60 *μ*m), but collagen shrinkage and remodelling—the 2 factors most likely responsible for the long-term clinical improvements seen after resurfacing—require 1 to 2 additional passes. When dermal temperatures exceed 55°C to 62°C, there is disruption of interpeptide bonds leading to conformational changes within collagen's triple helical structure that shrink the moiety to one-third its normal length. The mechanisms of long-term collagen remodelling and neocollagenesis after resurfacing are not fully known. However, it is believed that these effects result from thermal desiccation with concomitant collagen shrinkage [79, 80].

The advantages of CO_2_ laser include a reduced healing time, an infrequent need for anaesthesia, reduced thermal damage, less bleeding, less inflammation, and minimal undesirable side effects (unaesthetic scarring and dyschromic effects). These benefits must be considered in comparison with other available surgical techniques such as diathermocoagulation and traditional surgery which on the contrary necessitate frequent anaesthetics, a longer recovery period, a slower cicatrisation, and a high incidence of hypertrophic scars and keloids. Another relevant limitation of these two surgical treatments is its limited usage in many anatomically difficult areas such as the internal corner of the eyes, the ears, the alae of the nose, and the genitals, areas characterized by a high incidence of cutaneous damage upon using these techniques. The laser CO_2_ technique is preferred in patients with several or wide lesions, those with a pacemaker, or for patients who cannot be subjected to anaesthetics [75–78].

## 5. Conclusion

In this paper, we have stressed the efficacy and versatility of the “no-contact surgery” performed by the superpulsed CO_2_ laser and have witnessed that that this method is a valid alternative to surgery and/or diathermocoagulation for microsurgery of the soft tissues. The main benefit of the superpulsed system is the precise and adequate vaporization, its targeted thermal action on the lesions with minimal thermal damage of the adjacent areas which enables step-by-step visual assessment of the level reached, and an accurate calculation of the clinical “end point” by the operator, according to the principle of the “what you see is what you get.” The operator can decide whether only to vaporize the epidermis or to extend thermal damage even into the deeper derma.

 The technique guarantees the possibility of laser application without anaesthesia because the procedure itself is less painful and also because local anaesthesia could cause swelling and hinder the “visual feedback processing” in most cases. In our clinical practice, therefore, we do not use anaesthesia with powers lower than 1-2 W and in select cases suggest the application of a topical anaesthesia before treatment. This technique has few postoperative complications, and with appropriate patient compliance involving the application of topical antibiotics without scab removal (as this delays the healing process), the CO_2_ laser ensures rapid healing within one or two weeks. Finally, the possibility of intraoperative histologic and/or cytologic examination is another important advantage. The advent of fractional photothermolysis, initially introduced for non-ablative systems, has enabled the development of a new method that meets these demands perfectly, namely, the fractional microablative resurfacing with CO_2_ laser for treating wrinkles and damage caused by skin chronoaging, photoaging, or other environmental damages.

 These findings in essence make the CO_2 _ laser the most frequently used in our dermatologic clinical practice, especially for the treatment of aesthetic diseases. The treatment of wrinkles and scars using fractional lasers by contrast produces results that could create disappointing results in the short and long term. It must also be mentioned that the data obtained in our department are sometimes not consistent with those published in the literature, stressing the concept that multicentric studies oriented to the harmonization of the methods and patient selection are mandatory.

## Figures and Tables

**Table 1:** Treated lesions.

Benign epidermal tumours Seborrheic keratoses Follicular inverse keratoses Epidermal and sebaceous nevi	35.720 75 5706	Frequency: 10 HZ Power: 0.5–3 W
Benign pilar and sebaceous tumours Facial Milia Trichoepitheliomas Sebaceous adenoma Small sebaceous cysts	12.230 2.570 8.040 425	Frequency: 10 HZ Power: 0.5–1 W

Benign tumours of eccrine glands Syringomas	1235	Frequency: 10 HZ Power: 0.5–1 W

Malign epidermal tumours Basal Cell carcinoma (Bcc) Superficial (also extensive) Nodular (***<***1 cm diameter)	2505	Frequency: 10 HZ Power: 0.5–5 W

Viral lesions Warts Acuminate condylomas Squamous papillomas Oral cavity	12.235 8.503 58	Frequency: 10 HZ Power: 0.5–3 W For Plantar Warts: Continuous Mode (Power 10–15 W)

Dermal hypertrophy Pendulous fibromas Skin neurofibromas	45.023 150	Frequency: 10 HZ Power: 0.5–1 W

Scars Acne, surgical, traumatic, post-chickenpox	7.502	Frequency: 10 HZ Power: 0.5–1 W

Fatty accumulation Xanthelasmas	7.234	Frequency: 10 HZ Power: 0.5–1 W

Facial dermatosis Rhynophymas (glandular type) Otophyma	506 35	Frequency: 50–100 W Power: 6-7 W (Superpulse or Continuous Mode)

Precancerous Actinic keratoses Actinic cheilitis Leukoplakia	25.213 807 503	Frequency: 10 HZ Power: 0.5−3 W

Others Favre-Racouchot's disease Pringle-Bourneville's disease Chondrodermatites nodularis helicis	124 304 432	Frequency: 10 HZ Power: 0.5–1 W
